# The Lifetime Prevalence of Non‐Suicidal Self‐Injury in Children and Adolescents With Eating Disorders—A Systematic Review and Meta‐Analysis

**DOI:** 10.1002/erv.3158

**Published:** 2024-11-30

**Authors:** Marieke Meier, Katrin Jansen, Hannah Vertgewall, Laurence Claes

**Affiliations:** ^1^ Institute of Psychology University of Muenster Muenster Germany; ^2^ Faculty of Psychology and Educational Sciences KU Leuven Leuven Belgium; ^3^ Faculty of Medicine and Health Sciences (CAPRI) University of Antwerp Antwerp Belgium

**Keywords:** adolescents, eating disorders, meta‐analysis, non‐suicidal self‐injury, prevalence

## Abstract

**Objective:**

Eating disorders (EDs) and non‐suicidal self‐injury (NSSI) are both phenomena with onset in adolescence. Their co‐occurrence is associated with higher symptom severity and an elevated risk of suicide. In this meta‐analysis, we examine the lifetime prevalence of NSSI in youth with EDs.

**Methods:**

We searched PsycInfo, PubMed and previously published systematic reviews for studies reporting on lifetime NSSI prevalence among children and adolescents (19 years or younger) with an ED (anorexia nervosa, bulimia nervosa, binge eating or other specified feeding and EDs) published until June 2024. A generalized linear mixed model meta‐analysis was performed to estimate the pooled prevalence. Meta‐regressions and multivariate meta‐analyses were conducted to estimate separate prevalence rates based on ED diagnosis and care frame (e.g., inpatient vs. outpatient), respectively.

**Results:**

Fifteen studies comprising 3311 children and adolescents were included. Pooled lifetime NSSI prevalence across all ED diagnoses was 34.2% [CI: 27.5%–41.7%]. Heterogeneity was large (*I* ^2^ = 93.8%). Lifetime NSSI prevalence rates were significantly higher for participants with bulimia nervosa (53.6%) and those with anorexia nervosa binge‐eating/purging type (51.9%) than for participants with anorexia nervosa restrictive type (15.8%).

**Discussion:**

The small number of studies and the large heterogeneity limit the conclusiveness of this meta‐analysis. Results suggest an even higher prevalence of lifetime NSSI in adolescents with an ED than in adults with an ED. The results support previous findings indicating higher prevalence rates of NSSI for EDs associated with binge eating and purging behaviours than for restrictive EDs.

**Summary**

- This is the first study exclusively reviewing and synthesizing studies on lifetime prevalence of non‐suicidal self‐injury (NSSI) in youth with eating disorders (EDs). Despite the scarce body of available literature, we were able to include 15 studies (*n* = 3311) after an extensive literature research.
- About one third of children and adolescents with any ED has engaged in NSSI at least once in their life. Half of those with binge‐purging symptomatology have engaged in NSSI at least once.
- Assessment methods of NSSI and EDs varied immensely. We discuss potential implications for future research and give recommendations for clinical practice.

## 1 Introduction

Eating disordered (ED) behaviours and non‐suicidal self‐injury (NSSI) often co‐occur and are often used to cope with subjective distress or personal problems (Claes and Vandereycken 2007a). NSSI is defined as the intentional, self‐inflicted damage to one's own body tissue without suicidal intent and for purposes not socially sanctioned (Selby, Joiner, and Ribeiro 2014; Claes and Vandereycken 2007a; Nock 2010). Examples of NSSI include cutting, biting, scratching, hitting, or burning oneself. ED behaviours are usually associated with concerns about shape and weight, or preoccupation with food such as dietary restraint, self‐induced vomiting, or binge eating. The similarities and differences between NSSI and ED behaviours (e.g., functionality) are the topic of ongoing research with a special emphasis on their relationship in adolescence.

Both NSSI and EDs commonly emerge during adolescence (Cipriano, Cella, and Cotrufo 2017; Volpe et al. 2016), and are especially prevalent then (Cipriano, Cella, and Cotrufo 2017; Smink et al. 2014) with increasing incidence rates for NSSI and DE for female youth since the COVID‐19 pandemic. Specifically, in a large population‐based study in the UK, the incidence rates for EDs were 42% and for self‐harm 38% higher than predicted between March 2020 and March 2022 for girls aged 13–16 years (Trafford et al. 2023). NSSI prevalence rates range from 6.2% in preadolescent children (see meta‐analysis comprising 58 studies with community samples by Liu et al. 2022) to a mean lifetime prevalence in adolescents of 18% (see systematic review comprising 52 studies in community or school settings by Muehlenkamp, Claes, et al. 2012). In a large community cohort study, up until the age of 19, 5.7% of girls and 1.2% of boys had met criteria for an ED diagnosis according to the fifth edition of the Diagnostic and Statistical Manual (DSM‐5; American Psychiatric Association 2013), such as anorexia nervosa (AN), bulimia nervosa (BN) and binge eating disorder (BED; Smink et al. 2014).

Both NSSI and EDs are associated with significant threat for physical and mental health. They often co‐occur with other mental disorders (see for NSSI Kiekens et al. 2021; see for EDs Udo and Grilo 2019). NSSI and EDs are associated with suicidal ideation, future suicide attempts and increased risk of death by suicide (Ribeiro et al. 2016; Anestis, Khazem, and Law 2015; Smith et al. 2019; Mandelli et al. 2019). The co‐occurrence of EDs and NSSI is associated with more general psychopathology and higher functional impairment (Claes, Vandereycken, and Vertommen 2003; Muehlenkamp, Peat, et al. 2012; Washburn et al. 2023) as well as greater risk for suicide (Brausch and Perkins 2018; Pérez et al. 2019). Moreover, individuals who engage in both NSSI and ED behaviours, especially bingeing or purging, tend to make use of a larger variety of NSSI methods (Sorgi et al. 2021; Muehlenkamp et al. 2011), which is associated with a persistent course of NSSI and more suicidal behaviour (Turner et al. 2013; Anestis, Khazem, and Law 2015; Kiekens et al. 2017) as well as greater ED severity (Yiu et al. 2015).

So far, there are three meta‐analyses investigating the prevalence of NSSI in EDs (Cucchi et al. 2016; Kirkpatrick et al. 2023; Amiri and Khan 2023), and one meta‐analysis comparing the prevalence of NSSI in participants with EDs as well as in both healthy participants and psychiatric controls without eating disorder symptoms (Sohn et al. 2023).

Pooled prevalence rates of NSSI ranged from 27.3% (Cucchi et al. 2016) to 40.0% (Amiri and Khan 2023) with consistently higher rates among samples with binge‐purging symptomatology compared to restrictive symptomatology. While especially Kirkpatrick et al. (2023) included a comprehensive number of studies (*n* = 79), these studies mostly comprised adult samples (80% of included samples had a mean age of > 19 years).

One possible explanation for the high rate of co‐occurrence of EDs and NSSI can be that they share some common risk factors (e.g., trauma) and that in some cases they have the same function such as emotion regulation, punishing oneself etc. (Fox et al. 2019; Kline et al. 2023; S. B. Wang et al. 2021). Some of these shared risk factors (see conceptual model of Claes and Muehlenkamp 2014; also see Muehlenkamp et al. 2011) are especially salient during adolescence: With regard to emotional reactivity and negative mood intolerance, studies show that compared to children or adults, adolescents tend to experience more frequent negative emotions, tend to transition between emotional states more rapidly (Larson et al. 2002; Weinstein et al. 2007), and tend to have greater difficulties with emotion differentiation (i.e., separating affective experiences; Nook et al. 2018). Moreover, adolescents are especially sensitive to social evaluation by peers (Somerville 2013; Rodman, Powers, and Somerville 2017), which makes dysfunctional peer influence more puissant in adolescence. Lastly, while childhood maltreatment in general is a significant risk factor for both NSSI (Serafini et al. 2017) and EDs (e.g., Neumark‐Sztainer et al. 2000), in one study those who reported *recent* sexual abuse were almost seven times more likely to engage in NSSI than those with no history or a past history of sexual abuse (Tatnell et al. 2017).

Given the higher prevalence rates of ED and NSSI in adolescence compared to adulthood, the rising incidence rates of DE in youth, the severe consequences of NSSI and EDs on physical and mental development, and the particular vulnerability of some risk factors for NSSI and EDs in adolescence, it is relevant to synthesize studies investigating the prevalence rates of NSSI in youth with EDs to inform research, policy making and clinical practice. As previous meta‐analytic findings are not generalizable to children and adolescents with EDs, we aimed to synthesize lifetime prevalence rates of NSSI among children and adolescents with an ED. We decided to focus on lifetime prevalence as previous meta‐analyses have been collapsing different prevalence rates for synthesis (i.e., lifetime prevalence rates, period prevalence rates, or point prevalence rates), which greatly impedes comparability and interpretation. By narrowing the age of included samples to children and adolescents and assessing the lifetime prevalence of NSSI in this age group, we hope to determine NSSI prevalence closer to onset, thereby reducing biases (e.g., memory bias) that likely occurred when assessing NSSI in older samples.

Based on findings regarding differences in NSSI prevalence between ED diagnoses (e.g., Cucchi et al. 2016; Kirkpatrick et al. 2023), we aimed to explore NSSI prevalence separately per ED diagnosis (AN/BN/BED) and diagnostic subtype (AN restrictive [AN‐R] and binge‐purging subtype [AN‐BP]). Due to the clinical heterogeneity of EDs not otherwise specified (EDNOS) and other specified feeding and EDs (OSFED) diagnoses, this group was not considered for separate prevalence estimation. As Cucchi et al. (2016) found that prevalence rates were larger in samples with higher numbers of attempted suicides, we aimed to investigate differences in prevalence rates of lifetime NSSI depending on lifetime attempted suicide in younger samples. Moreover, as Cucchi et al. (2016) surprisingly reported significantly smaller prevalence rates of NSSI in samples with substance abuse, we aimed to explore differences in samples depending on lifetime substance abuse. Since mental disorders represent a risk factor for NSSI, it is more likely that NSSI occurs in clinical settings than in community settings (also see Bentley et al. 2015; Y. Wang et al. 2022). However, this was not yet investigated for children and adolescents, so that we decided to investigate differences in NSSI prevalence rates depending on the care frame. Lastly, as adverse childhood events (ACEs) represent a shared risk factor for NSSI and EDs, we included lifetime ACEs for moderator analysis. In summary, we aimed to explore differences in prevalence rates of lifetime NSSI in children and adolescents with EDs depending on (1) ED diagnoses, (2) care frame (3) lifetime substance abuse, (4) lifetime attempted suicide and (5) lifetime ACEs.

## 2 Methods

### 2.1 Preregistration and Study Protocol

We preregistered the aims and methods of this meta‐analysis in the PROSPERO database (CRD42023388740) and followed the PRISMA guidelines (Page et al. 2021). Deviations from the preregistered analyses are reported below.

### 2.2 Eligibility Criteria

Studies were considered eligible for inclusion if (1) they reported the prevalence rate of lifetime NSSI in a sample of individuals with EDs, (2) all individuals in the sample were diagnosed with AN, BN, BED, EDNOS, or OSFED, either by a clinician or by means of a validated screening instrument, (3) all individuals in the sample were aged 19 or below which is in accordance with the World Health Organization's (WHO 2022) definition of adolescence as the period of life between the ages 10 and 19 and if (4) the language of the article was either English or German. Studies were not eligible if (1) the assessment of self‐injury explicitly included behaviours exerted with suicidal intent, (2) they examined NSSI exclusively in the context of developmental disorders, (3) the primary ED diagnosis of any individual in the study's sample was rumination disorder, pica, or, avoidant restrictive food intake disorder (ARFID) and (4) they collected data on individuals for which the probability of NSSI was increased a priori (e.g., studies with a history of NSSI as inclusion criterion, or studies composed exclusively of patients with a comorbid borderline personality disorder) or only assessed one type of NSSI.

We did not set a minimum age as earliest onsets of EDs vary greatly between 6 and 12 years (Morris, Elliott, and Madden 2022; Stein, Chalhoub, and Hodes 1998) and because we did not want to further restrict the scarce body of available literature. Overall, these eligibility criteria, besides the described age limit, are in line with those employed in the meta‐analysis by Cucchi et al. (2016).

In order to investigate differences between ED diagnoses, we included samples that were composed of individuals with different diagnoses (mixed samples), samples comprising individuals with the same diagnosis (diagnosis‐specific samples), as well as samples restricted to a diagnostic subtype of AN (subtype‐specific samples). When studies reported data on a mixed sample as well as on subgroups with a specific ED diagnosis or diagnostic subtype, the mixed sample was considered eligible for the analysis of the overall prevalence, while the subgroup samples were considered eligible for the analysis of the prevalence for the specific ED diagnosis or diagnostic subtype. When a study reported only data from a diagnosis‐ or subtype‐specific sample, it was considered eligible for both analyses (i.e., analysis for overall prevalence and analysis for specific prevalences).

### 2.3 Study Selection

#### 2.3.1 Search Strategy

We searched the bibliographic databases PsycInfo (via EBSCOhost) and PubMed to retrieve all potentially relevant studies published until 25 June 2024. We applied the following search string:

(eating disorder* OR bulim* OR anorex* OR anorec* OR binge* OR ednos OR osfed^1^) AND (self‐harm* OR self‐injur* OR self‐mutilat* OR self‐destruct* OR parasuicid* OR self‐wound* OR cut* OR self‐cut* OR head‐bang* OR nail‐bit* OR hair‐pull* OR hitting OR picking OR skin‐pick* OR scratch* OR bruis* OR dermatillomania OR trichotillomania OR NSSI) AND (adolesc* OR child* OR youth OR teen* OR girls OR boys OR minors OR juvenile).

In addition to the database search, we assessed the reference lists of the meta‐analyses by Cucchi et al. (2016), Sohn et al. (2023), Amiri and Khan (2023) and Kirkpatrick et al. (2023) and the review by Kostro, Lerman, and Attia (2014). We also conducted a forward search based on these articles. With the aim to identify potentially relevant unpublished data, we searched the clinical trial registry clinicaltrials.gov for clinical trials on EDs and NSSI, and contacted the DGESS (Deutsche Gesellschaft für Essstörungen), a German interdisciplinary association of researchers and clinicians focusing on ED, as well as the Special Interest Group of Eating Disorders of the Association of Behavioural and Cognitive Therapies to ask for unpublished prevalence data on NSSI in children and adolescents with ED.

#### 2.3.2 Selection Process

All potentially eligible studies identified in the database search were exported to the literature management program CITAVI (Swiss Academic Software, 2021). Duplicates were removed. Eligibility of the remaining articles was assessed by one researcher (HV). Uncertainties regarding eligibility were discussed with the remaining authors. First, titles and abstracts were screened for eligibility and, if clearly not eligible, excluded without reading the full text. Then, the full texts of the remaining articles were obtained to further assess eligibility. Studies identified from sources other than PsycInfo or PubMed were screened separately by applying the same procedure.

For studies in which only a single exclusion criterion applied (e.g., maximum age above 19) and for which we suspected the data of the relevant subset to be eligible, the authors were contacted via e‐mail and asked for the respective data (e.g., prevalence of lifetime NSSI for the subsample of individuals aged 19 or below). Authors were also contacted via e‐mail when a study's eligibility remained unclear due to missing information in the manuscript.

### 2.4 Data Collection

Two researchers (H.V. and M.M.) independently extracted data from eligible articles. Data were extracted using a coding sheet, which was developed and adjusted by piloting on the first five eligible studies according to alphabetical order of author surnames. Inconsistencies in data extraction were solved by discussion among H.V. and M.M.

Where available, the following variables were extracted: (1) study author(s), (2) study title, (3) publication year, (4) region of study, (5) sample size, (6) number of participants with a history of lifetime NSSI, (7) number of participants with AN/AN‐R/AN‐BP/BN/BED/EDNOS respectively, (8) number of male/female/nonbinary participants respectively, (9) median age/mean age/standard deviation of age/minimum age/maximum age of the sample, (10) care frame (inpatients/day‐patients/outpatients/community sample), (11) diagnostic criteria applied for ED assessment (e.g., DSM), (12) definition of NSSI (e.g., NSSI definition according to the DSM‐5 [NSSI‐D]) and (13) assessment of NSSI (instruments, items etc.).

Although information on the following variables was also extracted, they were not reported in a sufficient number of studies to be considered for further analysis: (1) number of participants with a lifetime history of attempted suicide, (2) number of participants with a lifetime history of substance abuse and (3) number of participants with a lifetime history of ACE.

### 2.5 Effect Measures and Synthesis Methods

The main outcome for this meta‐analysis was the prevalence of NSSI, that is, the number of individuals with a history of lifetime NSSI divided by the sample size. Random‐effects meta‐analysis was used to obtain an estimate of the overall prevalence of NSSI across ED diagnoses. Specifically, we used a generalized linear mixed model (GLMM). This model is based on the raw data instead of on study‐specific effect sizes, which is advantageous in meta‐analyses of binary data (Stijnen, Hamza, and Özdemir 2010; Hamza, van Houwelingen, and Stijnen 2008). To ascertain that our results did not depend on the choice of this model, we fitted conventional random‐effects models as an additional sensitivity analysis. Heterogeneity was assessed by calculating prediction intervals (PIs; Riley, Higgins, and Deeks 2011) based on the quantiles of the standard normal distribution. A 95% PI indicates the region where 95% of the true prevalence rates are expected to fall in the hypothetical population of studies. In addition, we report the *I* ^2^ statistic (Higgins and Thompson 2002).

Further, we investigated whether heterogeneity was reduced when considering care frame as a moderator. To achieve this, we conducted a meta‐regression with care frame as a categorical predictor. Although five studies reported the lifetime prevalence of attempted suicide in their sample (our preregistered threshold regarding the number of studies necessary to conduct sub‐analysis), we deemed this number of studies insufficient for a meta‐regression for the following reason: In the five studies, either very low prevalence rates (below 5%, see Arnold et al. 2022; Islam et al. 2015; Ruuska et al. 2005) or rather large prevalence rates (around 20%, see Liang and Tseng 2011; Wiederman and Pryor 1996) were reported. Hence, it would not have been possible to evaluate whether a linear model for the logit prevalence rates is appropriate for the relationship. Moreover, although preregistered, substance abuse, ACEs and gender could not be included as potential moderators as not enough studies assessed these variables. Similarly, multicollinearity analyses that were originally planned, were impeded by the lack of reporting of almost all moderators of interest.

For the diagnosis‐specific analysis, we conducted a multivariate meta‐analysis based on the logit‐transformed prevalence rates. Although we had preregistered univariate meta‐analyses for the diagnosis‐specific subgroups, we opted for a multivariate analysis as some of the diagnosis‐specific samples were from the same study, and we suspected the true underlying prevalence rates of these samples to be more similar than the prevalence rates of samples from different studies. The model was fitted via restricted maximum likelihood (REML) estimation. As the analysis was based on only few studies, we decided to not estimate a fully unstructured variance‐covariance matrix for the random effects, but set the correlation between the true logit‐prevalence rates of samples from the same study to 0.5. In an additional sensitivity analysis, we explored whether setting the correlation to 0.1, 0.3, 0.7 or 0.9 led to a meaningful change in the results. Differences in the diagnosis‐specific prevalence rates were assessed using Wald‐type tests. The same approach was used for the subtype‐specific analysis.

We did not assess publication bias for the following reasons: First, prevalence of NSSI was usually not the main outcome of the studies that were eligible for this meta‐analysis, hence it was unlikely that publication of potentially eligible studies depended on this outcome. Second, the underlying assumption of methods to assess publication bias is that studies with a small precision that yield non‐significant results are lost in the publication process (Borenstein et al. 2009, chapter 30). However, for prevalence rates, it is usually not of primary interest whether they are significantly larger than zero. Hence, even if the NSSI prevalence was the main outcome of a study, its publication would probably not depend on its size or significance. We concluded that assessing publication bias in the way it is usually assessed in meta‐analyses is unlikely to provide any meaningful insights for the data at hand.

All analyses were conducted in R (R Core Team 2022) using the metafor package (Viechtbauer 2010). The results of all analyses were originally on the logit scale, but were converted to the prevalence scale to facilitate interpretation. Data and code for all analyses are publicly available at https://osf.io/847pq/.

## 3 Results

### 3.1 Study Selection

**FIGURE 1:**
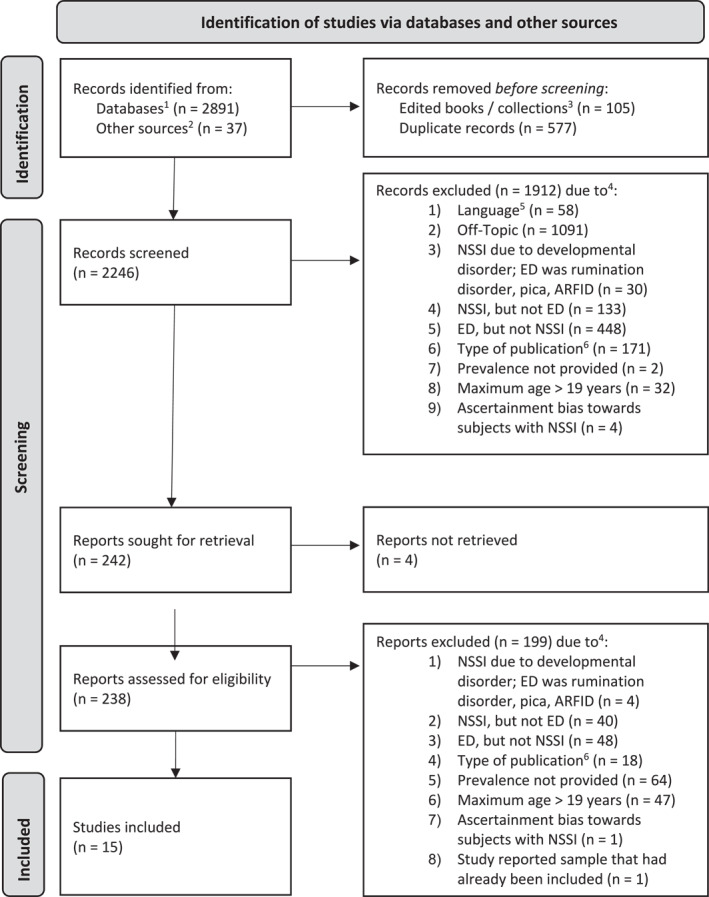
Flow diagram of study selection. Flow diagram adapted from Moher et al. (2009). ARFID = avoidant/restrictive food intake disorder; ED = eating disorder; NSSI = non‐suicidal self‐injury. (1) Databases: PsycInfo and PubMed. (2) Other sources: primarily forward and backward search of eligible studies and reviews. (3) Edited books were sorted out, only respective contributions were screened. (4) The exclusion criterion counted here is the one mentioned first in the specified order. (5) Language not English or German. (6) Type of publication or method, for example reviews and case studies.

The results of the study selection process are visualized in a flow diagram in Figure 1. A total of 2928 studies were obtained from database searches and other sources. After eliminating duplicate records, edited books and collections, 2246 entries remained. After abstract screening, 242 records were sought for retrieval, of which 238 were assessed for eligibility. The main reasons for exclusion were that studies did not provide data on the lifetime prevalence of NSSI (*n* = 64), that the maximum age of the sample exceeded 19 years (*n* = 47), and that studies reported on a sample of participants with EDs, but did not consider NSSI (*n* = 48), or that they reported on a sample of participants with NSSI, but were not diagnosed with an ED (*n* = 40).

### 3.2 Characteristics of the Included Studies

In total, 15 studies were included in this meta‐analysis. The main characteristics of these studies are summarized in Table 1. Preliminarily, six eligible studies were identified (Arnold et al. 2022; Buelens et al. 2020; Dzombak et al. 2020; Parker and Ricciardi 2019; Ruuska et al. 2005; Wiederman and Pryor 1996). The study by Dzombak et al. (2020) was included after correspondence with authors had confirmed that the age range, which was not reported in the paper, was 8–18 years. For this study, only part of the full sample was considered eligible as the subsample of ARFID patients had to be excluded. Concerning the study by Ruuska et al. (2005), only the AN subsample could be considered, as the maximum age of the full sample exceeded 19 years. Nine additional studies were included after their authors had provided prevalence estimates for subsamples that met our eligibility criteria via personal communication (cf., Table 1).

**TABLE 1:** Characteristics of eligible studies.

	Study	Country	*n*	Care frame	Age (years)	Mean (age)	% Male	ED diagnoses	Diagnostic ED	Diagnostic NSSI	% Lifetime NSSI
1	Arnold et al. (2022)	Germany	382	IN	9–18	15.4^a^	2.9	AN, BN	ICD‐10	Chart review	21.5
2	Buelens et al. (2020)^b^	Belgium	189	IN	14–17	15.93	0	AN, BN	DSM‐5	SIQ‐TR	59.8
3	Depestele et al. (2017)^b^	Belgium	42^c^	IN	14–19^c^	—	0	AN, BN, EDNOS^d^	DSM‐IV	SIQ‐TR	64.3^c^
4	Dzombak et al. (2020)^e^	USA	142	DAY	—	14.35	9.9	AN, BN, OSFED	DSM‐5	Chart review	43.0
5	Islam et al. (2015)^b^	Spain	322^c^	IN	14–19^c^	18.17^a^	—	AN, BN, BED, EDNOS^d^	DSM‐IV‐TR	Interview	30.1^c^
6	Liang and Tseng (2011)^b^	Taiwan	78^c^	OUT	13–19^c^	17.34^a^	0	AN, BN, EDNOS	DSM‐IV	Single question^f^	39.7^c^
7	Miyawaki et al. (2018)^b^	Japan	90^c^	OUT	13–19^c^	—	0	AN, BN	DSM‐5	Interview	36.7^c^
8	Olatunji et al. (2015)^b^	USA	955^c^	IN	13–19^c^	—	0	AN, BN, EDNOS^d^	DSM‐IV‐TR	Interview	39.4^c^
9	Parker and Ricciardi (2019)	Australia	367	COM	^g^	—	24.5	—	SCOFF	Single question^f^	26.4
10	Reas, Wisting, and Lindvall Dahlgren (2023)^b^	Norway	307^c^	COM	—	—	—	—	EDE‐QS	DSHI	41.7^c^
11	Ruuska et al. (2005)^h^	Finland	34	OUT	14–18.7	16.2	0	AN	ICD‐10	Single question^f^	14.7
12	Scheiner et al. (2022)^b^	Germany	117^c^	COM	—	—	—	—	SEED	DSHI‐9	14.5^c^
13	Vieira et al. (2017)^b^	Portugal	100^c^	OUT	14–19^c^	16.94^a^	0	AN, BN	DSM‐5	Interview	34.0^c^
14	Wiederman and Pryor (1996)	USA	117	OUT	12–17	15.44	0	AN, BN	DSM‐III‐R	DSED‐R	21.4
15	Wohlfahrt, Strack, and Reich (2022)^b^	Germany	69^c^	OUT	13–19	—	0	AN, BN	ICD‐10^a^	Single question^f^	43.5^c^

Abbreviations: AN = anorexia nervosa, BED = binge eating disorder, BN = bulimia nervosa, COM = community sample, DAY = day patients, DSED‐R = Diagnostic Survey for Eating Disorders—Revised (Johnson 1985), DSHI = Deliberate Self‐Harm Inventory (Gratz 2001), DSHI‐9 = Deliberate Self‐Harm Inventory, adapted for adolescents (Lundh, Karim, and Quilisch 2007), DSM = Diagnostic and Statistical Manual of Mental Disorders (R: revised; TR; text revision), ED = eating disorder, EDE‐QS = Eating Disorder Examination—Questionnaire Short (Gideon et al. 2016), EDNOS = eating disorder not otherwise specified, IN = inpatients, NSSI = non‐suicidal self‐injury, OSFED = other specified feeding and eating disorder, OUT = outpatients, SCOFF = screening tool for Eating Disorders (Morgan, Reid, and Lacey 1999), SEED = Short Evaluation of Eating Disorders, screening instrument for main symptoms of AN and BN (Bauer et al. 2005), SIQ‐TR = The Self‐Injury Questionnaire‐Treatment Related (Claes and Vandereycken 2007a, 2007b).

^a^ Information retrieved from personal correspondence with authors.

^b^ Study's main sample does not meet eligibility criteria; data given in the table refer to adjusted sample recalculated by the authors to fit eligibility criteria regarding age range or ED diagnosis.

^c^ Information retrieved from personal correspondence with authors; specific for recalculated sample; data listed here differs from published data for full sample.

^d^ Diagnoses examined in the full sample; no information on whether all diagnoses are present in the recalculated sample.

^e^ Only part of the full sample was included, as the subsample of ARFID‐patients had to be excluded.

^f^ Patients self‐reported whether they had engaged in NSSI, for example, ‘Have you ever tried to physically hurt yourself?’.

^g^ No age range provided—study was included as sample consists of high school students.

^h^ Only AN sample was eligible; full sample was not included as maximum age exceeded 19 years.

Overall, this meta‐analysis includes seven studies that had not been included in the meta‐analysis conducted by Kirkpatrick et al. (Buelens et al. 2020; Parker and Ricciardi 2019; Reas, Wisting, and Lindvall Dahlgren 2023; Scheiner et al. 2022; Vieira et al. 2017; Wiederman and Pryor 1996; Wohlfahrt 2022) and 12 that were published after Cucchi et al.'s analysis (2016) and were not included in their analysis.

Across the included studies, definition and assessment of NSSI varied considerably. Most studies (*n* = 10) described the examined behaviour as non‐suicidal, although suicidal intent was only explicitly ruled out during assessment in one study (Reas, Wisting, and Lindvall Dahlgren 2023). No study assessed NSSI based on DSM‐5 criteria for NSSID. While five studies used questionnaires such as the SIQ‐TR (Self‐Injury Questionnaire—Treatment Related; Claes and Vandereycken 2007b) and the DSHI (Deliberate Self‐Harm Inventory; Gratz 2001; Lundh, Karim, and Quilisch 2007), four studies used a single question in form of a questionnaire (‘Have you ever tried to physically hurt yourself?’). Even those studies that assessed NSSI with an interview often used a one‐item screener and only followed up if patients answered in the affirmative.

### 3.3 Meta‐Analytic Results

#### 3.3.1 Prevalence of NSSI Across Diagnoses

**FIGURE 2:**
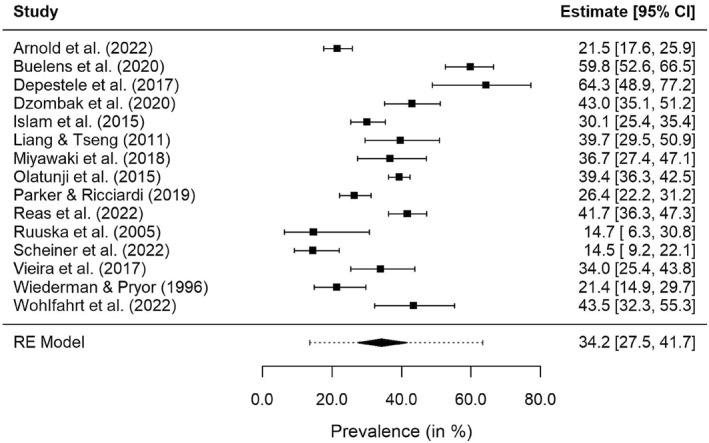
Forest plot for the main meta‐analysis. The dotted line indicates the 95% PI.

The analysis of the prevalence of NSSI across ED diagnoses was based on 15 studies with a total number of 3311 subjects. Nine of the studies were conducted in Europe, three in the USA, two in Asia and one in Australia. The proportion of females ranged from 75.5% to 100%. In nine studies all subjects were female. Regarding the care frame, the data consisted of five outpatient samples (*n* = 1890), six inpatient samples (*n* = 488), three community samples (*n* = 791) and one day patient sample (*n* = 142). Prevalence rates of the individual samples, along with 95% CIs, are depicted in Figure 2. The random‐effects meta‐analysis yielded an overall prevalence estimate of 34.2% (95% CI: 27.5%–41.7%). Heterogeneity was large, as indicated by the 95% PI which ranged from 13.6% to 63%, and the magnitude of *I* ^2^ (93.8%). Similar results were obtained in the sensitivity analysis using the conventional random‐effects model for meta‐analysis instead of the GLMM.

#### 3.3.2 Moderator Analyses

**FIGURE 3:**
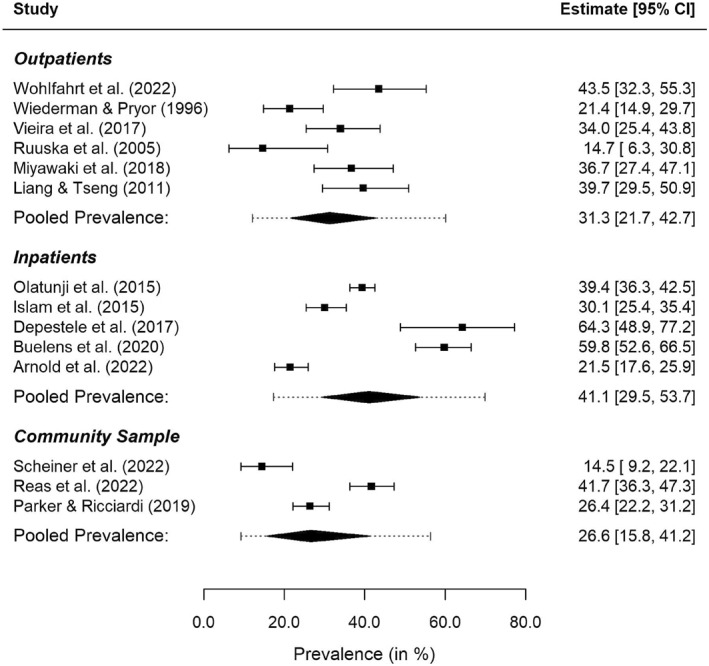
Forest plot for the meta‐regression with care frame as a predictor. Dotted lines indicate the 95% PIs obtained for the respective subgroup.

As there was only one sample with day patients, the meta‐regression with care frame as the predictor was conducted based only on inpatient, outpatient and community samples and thus included a total number of 14 samples (*n* = 3169). Figure 3 shows a forest plot along with the meta‐analytic results per care frame. There was no significant reduction in heterogeneity when considering care frame as a moderator ($Q_{M}=2.68,df=2,p=0\;.262$). The estimated average prevalence of NSSI was smallest for community samples (26.6%, 95% CI: 15.8%–41.2%, 95% PI: 9.2%–56.4%), followed by outpatient samples (31.3%, 95% CI: 21.7%–42.7%, 95% PI: 12.1%–60.1%), and was largest for inpatient samples (41.1%, 95% CI: 29.5%–53.7%, 95% PI: 17.3%–69.9%). However, the differences between these prevalence rates were not significant while residual heterogeneity was large (*I* ^2^ = 92.5%). The estimates of the average prevalence rates obtained in the sensitivity analysis using conventional random‐effects meta‐regression were similar, but interval estimates (both CIs and PIs) tended to be slightly wider (results not shown).

#### 3.3.3 Diagnosis‐Specific Analysis

**FIGURE 4:**
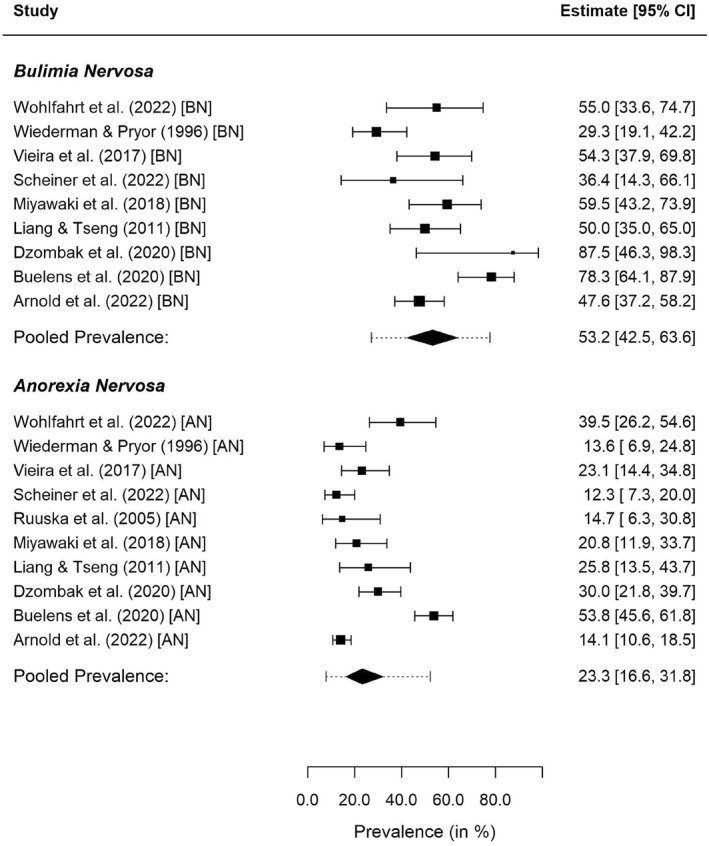
Forest plot for the diagnosis‐specific meta‐analysis. Dotted lines indicate the 95% PIs obtained for the respective subgroup.

Ten studies provided data for samples of subjects with AN, and nine of these studies also provided data for samples of subjects with BN. None of the studies reported data of a sample consisting only of subjects with BED. Prevalence rates of the individual samples, along with 95% CIs, are depicted in Figure 4. For AN, multivariate meta‐analysis yielded an estimated average prevalence of lifetime NSSI of 23.3% (95% CI: 16.6%–31.8%) with substantial residual heterogeneity (95% PI: 7.8%–52.2%). For BN, we obtained an estimated average prevalence of NSSI of 53.2% (95% CI: 42.5%–63.6%), again with substantial residual heterogeneity (95% PI: 27.1%–77.6%). The difference between the average prevalence rates of NSSI in AN versus BN samples was significant ($Q_{M}=27.84,df=1,p\;<\;0.001$). These findings were robust to the value of the correlation between the true logit prevalence rates specified in the analysis.

#### 3.3.4 Subtype‐Specific Analysis

**FIGURE 5:**
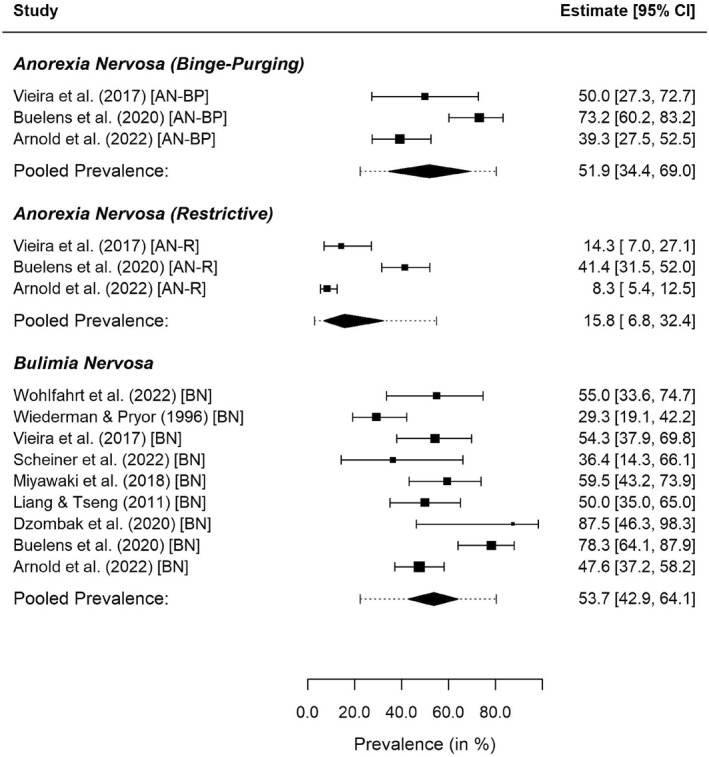
Forest plot for the diagnostic subtype‐specific meta‐analysis. Dotted lines indicate the 95% PIs obtained for the respective subgroup.

Three studies provided data for samples of subjects with AN‐R and AN‐BP. In order to be able to statistically compare the estimated average prevalence rates of NSSI for AN‐R and AN‐BP samples to that of BN samples, we also included the nine BN samples in the subtype‐specific analysis. Prevalence rates of the individual samples, along with 95% CIs, are depicted in Figure 5. For AN‐R, multivariate meta‐analysis yielded an estimated average prevalence of NSSI of 15.8% (95% CI: 6.8%–32.4%) with substantial residual heterogeneity (95% PI: 2.8%–54.9%). For AN‐BP, the estimated average prevalence was considerably larger at 51.9% (95% CI: 34.4%–69.0%), again with substantial residual heterogeneity (95% PI: 22.2%–80.3%). The results for BN deviated only slightly from those obtained in the diagnosis‐specific analysis (53.7%, 95% CI: 42.9%–64.1%, 95% PI: 27.5%–78.0%). The differences between the average prevalence rates were significant for AN‐BP versus AN‐R (*p* < 0.001) and for BN versus AN‐R (*p* < 0.001), but not for AN‐BP versus BN (*p* = 0.855). Although statistical inference was unaffected by the value of the correlation between the true logit prevalence rates specified in the analysis, the estimates of the average prevalence rates and the estimated between‐study variances varied somewhat across correlations, potentially due to the number of samples for the categories AN‐R and AN‐BP being small.

## 4 Discussion

This meta‐analysis is the first to synthesize lifetime prevalence rates of NSSI exclusively in children and adolescents with EDs. Overall, we pooled data from 15 studies, mostly conducted in Europe, and a total of *n* = 3311 (mostly female) participants between the age of 9 and 19. Notably, data of 9 studies was obtained through personal communication with the authors as the original studies reported on wider age ranges. We estimated a lifetime prevalence rate of 34.3% (min = 14.5% to max = 64.3%) across studies, which exceeds the reported 27.3% in Cucchi et al.'s (2016) analysis of predominantly adult samples with EDs and is similar to the not specified prevalence of 34.6% in the most recent and comprehensive meta‐analysis comprising mostly adult samples (Kirkpatrick et al. 2023).

The estimated prevalence of lifetime NSSI among youth with EDs was greatest in inpatient samples (41.1%), followed by outpatient samples (31.13%) and smallest in community samples (26.6%) which broadly aligns with Cucchi et al.'s (2016) findings and is reasonable given potentially more comorbidity in inpatient settings which increases the risk for NSSI (Bentley et al. 2015; Y. Wang et al. 2022). Notably, the lifetime prevalence of NSSI in community settings with EDs still clearly exceeds the prevalence of NSSI of adolescents without EDs (18%; Muehlenkamp, Claes, et al. 2012) which underlines the increased risk for NSSI in ED samples, irrespective of care frame.

Overall, inter‐study heterogeneity was high for the overall analysis as well as for subtype analyses and comparable to other meta‐analyses in the field (Kirkpatrick et al. 2023: *I* ^2^ = 98.7%; Cucchi et al. 2016: *I* ^2^ = 88.7%; Amiri and Khan 2023: *I* ^2^ = 97.4%). Interestingly, the inclusion of moderators did not reduce heterogeneity which complicates identifying further causes for heterogeneity. While heterogeneity can arise from methodological, clinical and statistical differences (Purgato and Adams 2012), we suggest that the differences in the way NSSI was assessed (interviews, single‐item screeners) contributed to substantial heterogeneity irrespective of sample characteristics. For future research, we propose that the assessment of NSSI should be standardized as a first step toward more comparable studies.

Our results indicate significantly greater lifetime prevalence rates of NSSI in individuals with BN (53.2%) than those with AN (23.3%). Consistently, we found significantly higher rates for those with binge‐purging symptomatology (BN, AN‐BP) than those with restrictive symptomatology (AN‐R) which mirrors previous meta‐analytic research in older samples (Cucchi et al. 2016, Kirkpatrick et al. 2023; Amiri and Khan 2023) and studies pointing to greater emotion regulation difficulties in adolescents with binge‐purging than restrictive symptomatology (Weinbach, Sher, and Bohon 2018). Moreover, this difference can be interpreted against the backdrop of studies suggesting that while NSSI, bingeing and purging are primarily maintained by automatic negative reinforcement (e.g., alleviation of tension; Wedig and Nock 2010), restrictive eating may be primarily maintained by automatic positive reinforcement (e.g., feelings of control; S. B. Wang et al. 2021; Nordbø et al. 2006). Thus, NSSI and binge/purging behaviours may co‐occur more often because individuals use different behaviours when trying to alleviate negative emotional states.

Despite its strengths such as an extensive literature search, the use of lifetime prevalence instead of a conglomerate of different prevalences, the inclusion of non‐published data, dual coding of all studies, and the computation of sensitivity analyses, our meta‐analysis has limitations. First, the number of eligible studies was small and potentially relevant moderators (history of substance abuse, suicide attempts, or ACEs) could not be investigated as they were not frequently assessed. Second, studies suggest that prevalence rates are greater when behaviour checklists instead of single‐item questions and a larger number of specific NSSI methods are used (see meta‐analysis by Swannell et al. 2014, also see Robinson and Wilson 2020). Given that most included studies assessed NSSI using single‐item screeners (even if interviews were conducted), prevalence rates of NSSI have potentially still been underestimated. Third, samples with BED, males, or preadolescent children were only rarely examined or completely missing from the analysis, which impedes generalizability beyond female adolescents with EDs. Kirkpatrick et al.'s (2023) analysis suggests lowest prevalence rates of NSSI in samples with BED, but we were unable to confirm this for children and adolescents due to a lack of studies, which aligns with the general lack of studies on BED among children and adolescents (see Kjeldbjerg and Clausen 2023), potentially given diagnostic difficulties of BED in youth (see Bohon 2019). Fourth, while the coding sheet was independently rated and corrected, initial study eligibility was assessed by only one rater and questions were discussed within the team. This presents a limitation as thereby studies may have been overlooked.

Considering that approximately one third of adolescents with ED have engaged in NSSI, more research is warranted on causes of co‐occurrence, especially regarding shared motives and functions of EDs and NSSI. Furthermore, this study underlines the necessity for health care professionals to assess NSSI (preferably with a behaviour checklist) when they treat youth with EDs. This is particularly important in light of that NSSI is only rarely disclosed (Rowe et al. 2014), ED behaviours are commonly associated with feelings of shame and guilt (Blythin et al. 2020; O'Loghlen, Grant, and Galligan 2022), and that providers screen for NSSI in less than half of patients with EDs (Peebles, Wilson, and Lock 2011). Earlier detection and treatment (e.g., skills training to replace self‐harming strategies to alleviate pain; Linehan 2014) could help curb consequences of comorbid NSSI in ED, most notably the increased risk of suicide.

## Conflicts of Interest

The authors declare no conflicts of interest.

## Data Availability

Data and code for all analyses are available at on OSF (https://osf.io/847pq/).

## Endnotes

^1^
‘osfed’ was not included in the preregistered PROSPERO search string, but was included later during the review process and during the updated literature search.
